# Morphological spectrum of endometrium with thyroid hormone profile in infertile female population of Khyber Pakhtunkhwa province of Pakistan

**DOI:** 10.12669/pjms.36.5.1791

**Published:** 2020

**Authors:** Sabeen Nasir, Sara Ziaullah, Sadaf Alam, Muhammad Mumtaz Khan

**Affiliations:** 1 Dr. Sabeen Nasir, MBBS, M. Phil., Department of Pathology, Peshawar Medical College, Peshawar, Pakistan; 2 Dr. Sara Ziaullah, MBBS, M. Phil., Associate Professor, Department of Pathology, Peshawar Medical College, Peshawar, Pakistan; 3 Dr. Sadaf Alam, MBBS, M. Phil., Associate Professor, Department of Pathology, Peshawar Medical College, Peshawar, Pakistan; 4 Dr. Muhammad Mumtaz Khan, MBBS, M. Phil., Department of Pathology, Peshawar Medical College, Peshawar, Pakistan

**Keywords:** Endometrium, Infertility, Thyroid hormones

## Abstract

**Objectives:**

To study the morphological spectrum of endometrial changes with the thyroid hormone levels in infertile women.

**Methods:**

This cross sectional study was conducted at Department of Pathology, Peshawar Medical College, Health Care Centre and Madina Medical Laboratory, Peshawar from April 2013 to August 2013. Total 160 cases of infertile women were included in the study. Biopsies were taken on 22-23rd day of menstrual cycle. Statistical Analysis was performed using SPSS version 19 statistical program. Difference between endometrial findings of patients with normal thyroid profile and abnormal one were analyzed for statistical significance using Chi square test. Probability values p ≤ 0.05 were considered statistically significant.

**Results:**

In our study, majority infertile women were euthyroid (80%), followed by hyperthyroid (18%) and only two% to be hypothyroid. The correlation of endometrial morphology with thyroid hormones levels turned out to be insignificant. While correlating histological details of endometrial biopsy with thyroid hormone status, we found significant association of leukocytic infiltrate with thyroid hormone levels.

**Conclusion:**

This study shows that thyroid hormones may have role in early leukocytic infiltration into stroma, and a possibility of immune modulation by altered thyroid hormones in causing infertility.

## INTRODUCTION

Infertility is a growing problem affecting 8-12% couples of reproductive age group.^1^ It is defined as the failure to establish a clinical pregnancy after 12 months of regular and unprotected sexual intercourse. Primary infertility refers to a condition in which a woman has never been diagnosed with a clinical pregnancy, while in secondary infertility, a woman is unable to establish a clinical pregnancy after previously been diagnosed with a clinical pregnancy.^2^

There are certain obstacles in true estimation of prevalence of infertility i.e. lack of consensus on definition, uniform tools for diagnosis, underreporting of infertile individual and couples. However, the available data shows that prevalence of infertility differs among various geographical regions. It also varies from country to country and from region to region within the same country.^3^ In developed countries the prevalence of infertility ranges between four percent to 16.7%. In less developed countries percentage ranges between seven percent to nine percent.^4^ The global prevalence of primary infertility is 2%, while that of secondary infertility it is about 10.5%.^3^Pakistan is located in South Asia which is high infertility prevalence region.^5^ In Pakistan, the estimated prevalence of infertility is 21.9%, of which primary infertility is four percent and secondary infertility is 18.4%.^6^

There are many causes of infertility in females. They can be broadly classified as anatomical and hormonal. Anatomical causes include tubal blockage (14-15%), uterine (16-17%) and cervical (15%) causes.^7^

Hormonal abnormalities contribute to infertility in women. Besides the hormones of hypothalamic-pituitary-gonadal axis, which mainly regulates the reproductive system, role of thyroid hormones and prolactin cannot be overlooked. Abnormal thyroid hormone/prolactin levels may cause menstrual disturbances, which can lead to female infertility.^8^,^9^ Thyroid hormone receptors are also found in both endometrial glands and stroma.^10^ This further supports the idea that thyroid hormone may have role in bringing out morphological changes in endometrium, necessary for establishment of pregnancy. The prevalence of abnormal thyroid hormones levels in infertile women is variable globally. Hyperthyroidism in such women ranges from 2% to 5% while hypothyroidism in infertile women ranges from 2% to 8%.^8^,^11^,^12^

Human endometrium is a steroid hormone responsive tissue, whose cellular components respond to cyclic changes in circulating ovarian derived estradiol and progesterone. The endometrium undergoes precisely defined morphological changes until a receptive endometrium is developed.^13^ Different morphologic patterns of endometrium usually associated with infertility include disordered proliferative endometrium, hyperplasia, luteal phase defect and endometritis.^14^

Much work has been done regarding morphological changes in endometrium with steroid hormone dysfunctions. However, studies correlating morphological changes in endometrium with thyroid hormone disorders are lacking. Morphological spectrum in response to changes in thyroid profile have been studied in experimental animals,^15^,^16^ however no such work has been carried out in infertile women specifically in our setup. Therefore, the objective of our study was to observe the effects of blood thyroid levels on endometrial morphology of infertile women with an aim to rule out thyroid abnormalities as a cause of infertility.

## METHODS

This cross sectional study was conducted at Department of Pathology Peshawar Medical College, Health Care Centre and Madina Medical Laboratory and Diagnostic Center Peshawar from April 2013 to August 2013. After approval by Institutional Review Board (Ref No. Prime/IRB/2019-188) 160 cases of infertile women were recruited. Those females with normal laparoscopic findings and normal semen analysis of their spouse were included in the study through consecutive sampling. Females with organic lesions on laparoscopy were excluded from study. Blood samples for thyroid function tests were obtained and biopsies were taken on 22-23^rd^ day of menstrual cycle and Hematoxylin & Eosin (H&E) stained slides were examined under light microscope. Endometrial biopsies showing secretory phase were considered normal. Estimation of serum levels of thyroid hormones (TSH, T3 and T4) were performed on Chemiluminescence Immunoassay Analyzer CLIA-IIS manufactured by Biomed Engineering, China. The reference values for thyroid hormones status were taken as TSH (0.35 to 5.3 µlU/mL), T4 (5.0 to 13.0 µg/dL), T3 (0.8 to 1.9 ng/mL). Values below/above were considered abnormal.

### Statistical analysis

It was performed using SPSS version 19 statistical program. Statistical results were presented as mean and standard deviation for continuous variables. Difference between endometrial findings of patients with normal thyroid profile and abnormal one were analyzed for statistical significance using Chi square test. Probability values p≤0.05 were considered statistically significant.

## RESULTS

This study consisted of 160 infertile women of reproductive age group. Out of these cases, 102 (63.75%) belonged to primary infertility group. Fifty-eight (36.25%) cases had secondary infertility. Majority of women (80%) had normal thyroid profile. Among those with abnormal thyroid profile, 29 (18%) patients were hyperthyroid and only 3(2%) patients turned out to be hypothyroid. The age range was from 16-42 years with a mean age of 28.13 years (SD±5.11).

The endometrial findings in euthyroid and hyperthyroid patients are shown in Tables I-II. While calculating p-value, we considered only hyperthyroid patients versus euthyroid patients, because hypothyroidism was found only in three patients.

**Table-I:** Morphological patterns of endometrium in Infertile women with Thyroid Hormone Status.

Sr. No.	Endometrial Morphology (N= 157)	Serum Thyroid Hormone Status				Value of Chi square	p-Value

		Euthyroid		Hyperthyroid			

		%	n	%	n		
1.	Secretory Phase (n= 101)	79	80	21	21	0.627	0.42
2.	Proliferative Phase (n= 08)	75	6	25	2	0.07	0.76
3.	Irregular Maturation (n= 12)	83	10	17	2	0.11	0.73
4.	Disordered Proliferative (n= 27)	93	25	7	2	2.58	0.10
5.	Simple Hyperplasia (n= 03)	67	2	33	1	1.91	0.16
6.	Complex Hyperplasia (n= 06)	83	5	17	1	0.05	0.8

**Table-II:** Histological details of endometrial biopsy and its correlation with thyroid hormone status.

	Abnormal	Normal	Value of Chi square	p-Value
	***Shape of Glands***			
Hyperthyroid	34	66	2.092	0.14
Euthyroid	45	55		
	***Lining epithelium***			
Hyperthyroid	34	66	0.345	0.55
Euthyroid	39	61		
	***Edema***			
Hyperthyroid	66	34	0.023	0.88
Euthyroid	68	32		
	***Decidualization***			
Hyperthyroid	41	59	1.381	0.24
Euthyroid	32	68		
	***Spiral arterioles***			
Hyperthyroid	48	52	2.010	0.15
Euthyroid	59	41		
	***Leucocytic infiltrate***			
Hyperthyroid	98	2	5.26	0.021
Euthyroid	89	11		

Morphological patterns of endometrium in infertile women with Thyroid Hormone status is shown in Table-I. The endometrial biopsy of 101 patients (64.33%) showed secretory phase, which is a normal finding at 22^nd^/23^rd^ day of menstrual cycle. The second major group was of disordered proliferative endometrium comprising of 27 patients, followed by irregular maturation of endometrium. The correlation of endometrial morphological patterns with serum thyroid hormones levels turned out to be insignificant.

Histological details of endometrial biopsy and its correlation with thyroid hormone status are shown in Table-II. The parameters that we considered included shape of the glands and type of their lining epithelia. The stromal details included stromal edema, decidualization, leukocytic infiltrate and thickness of spiral arterioles. Only association of leukocytic infiltrate to thyroid hormone levels was significant.

## DISCUSSION

For implantation of embryo and establishment of pregnancy, properly formed endometrial bed is mandatory. The required morphological changes are carried out by ovarian steroid hormones, estrogen and progesterone.^17^ However, as a general metabolic hormone the possible action of thyroid hormone in bringing different morphological changes at various phases of endometrial development cannot be ignored.^18^

Some local and foreign workers have published articles on relationship of altered thyroid hormone levels with infertility, but they lack in relating it to associated morphological changes in endometrium.^19^-^22^ Another group of authors considered morphological changes in endometrium of infertile women, without taking in account the thyroid hormone status of patients.^23^,^24^ However, such studies on animal subjects are present; they focus histological changes in endometrium only.^15^,^16^ Our study is unique in considering morphological patterns along with histological changes in endometrium.

The thyroid hormone levels of majority of infertile females in our study are within normal range (80%). These results are similar to the studies done in Peshawar and Faisalabad which show 94% and 62.2% of infertile women to be euthyroid.^19^,^25^ Likewise, studies done in India and Nepal show 75% of infertile patients were euthyroid.^12^,^26^ The frequency of hyperthyroid patients in our study is 18% while hypothyroid patients constitute only 2% of patients. This may not be surprising as hyperthyroidism is more prevalent in our region as compared to hypothyroidism.^27^,^28^ These findings also correlate with study done in Pakistan by Lalani S and in India by Kameswaramma K et al., while studies done in Nepal by Rijal B et al. and in Pakistan by Naib et al. shows high prevalence of hypothyroidism as compared to hyperthyroidism in infertile women.^12^,^19^,^29^,^30^

In our study we found that in majority of infertile women (64%), the morphological pattern of endometrium (secretory phase) coincides with dates, irrespective of thyroid hormone status. In remaining females (36%) the endometrium shows a variety of changes, including disordered proliferative endometrium, hyperplasia, irregular maturation etc. There was no significant difference between hyperthyroid and euthyroid groups. We couldn’t find any other study relating morphological changes in human endometrium with thyroid hormone derangements in infertility.^31^ However, there are studies available which can explain the reason for our findings. For example, some studies reveal that thyroid hormone may be synthesized locally in endometrial tissue and act in a paracrine fashion.^10^,^32^ In addition gonadal steroids can also modulate expression of thyroid hormone receptors in endometrium.^33^ Because of multiple mechanisms involved in thyroid hormones actions, it is not necessary that serum hormone levels may always be predictive of its availability and activity at cellular levels.^34^

Regarding histological details, we compared our findings with studies on animals, because no human study is present so far. According to our study, p-value is only significant for leukocytic infiltrate in endometrial stroma. This is in contrast to study done on rats by Lutsyk and Sogomonian which revealed heavy leucocyte infiltration of the endometrial stroma in hypothyroidism.^15^ However, some studies show that hyperthyroidism induce leucocytic proliferation.^35^

## CONCLUSION

This study shows that thyroid hormones may have role in early leukocytic infiltration into stroma, and a possibility of immune modulation by altered thyroid hormones in causing infertility.

### Recommendations

Further studies are recommended to find any possible role of thyroid hormones on endometrium at subcellular levels in infertile females. In addition, studies relating to the possible role of thyroid antibodies in causing infertility should be done.

### Author`s Contribution

**SN** conceived, designed and did data collection & editing of manuscript. Responsible and accountable for the accuracy or integrity of the work.

**SZ** did statistical analysis and manuscript writing.

**SA** did review and editing of manuscript.

**MMK** did review and final approval of manuscript.
